# Diabetes Insipidus Complicating Management in a Child with COVID-19 and Multiorgan System Failure: A Novel Use for Furosemide

**DOI:** 10.1155/2021/5942431

**Published:** 2021-08-04

**Authors:** Sara D. Gungor, Robert P. Woroniecki, Erin Hulfish, Katherine V. Biagas

**Affiliations:** ^1^Stony Brook Children's Hospital, 101 Nicolls Road, Health Sciences Tower Floor 11-040, Stony Brook, New York 11794, USA; ^2^Renaissance School of Medicine at Stony Brook University, 101 Nicolls Road, Stony Brook, New York 11794, USA

## Abstract

Judicious balance of fluids is needed for optimal management of acute respiratory distress syndrome (ARDS). Achieving optimal fluid balance is difficult in patients with disorders of fluid homeostasis such as diabetes insipidus (DI). There is little data on the use of Furosemide to aid in balancing fluid and electrolytes in patients with DI. Here, we present a critically ill 11-year-old female with developmental delay, septo-optic dysplasia, central DI, and respiratory failure secondary to COVID-19 ARDS. She required careful titration of a Vasopressin infusion in addition to IV Furosemide for successful management of fluid and electrolyte derangements. On admission, she demonstrated high-volume urine output with mild hypernatremia (serum sodium 156 mmol/L). Despite her maximum Vasopressin infusion rate of 8 mU/kg/hr, by day two of admission, she voided a total of 4 L resulting in severe hypernatremia (serum sodium 171 mmol/L). With continually high Vasopressin infusion rates, her overall fluid balance became increasingly net positive, although her hypernatremia persisted. Her ARDS continued to worsen. After 48 hours of the addition of intermittent Furosemide, successful diuresis along with resolution of hypernatremia was achieved. The combination of IV Furosemide with Vasopressin infusion resulted in tailored diuresis and more controlled titration of serum sodium levels than adjustment in Vasopressin and fluids alone. These results are in contradistinction to the published literature, which focuses on the use of thiazide diuretics in managing DI. This experience highlights the potential for loop diuretics to aid in establishing a desired fluid and electrolyte status in managing patients with both DI and ARDS.

## 1. Introduction

In the critically ill patient, delicate balance of fluids is needed to achieve optimal outcomes. In critically ill children, several studies demonstrate that those with an overall excess in fluid balance have been shown to have higher levels of both morbidity and mortality [1–3]. In mechanically ventilated children and adults with ARDS, high cumulative fluid balances have been shown to contribute to longer durations of intubation as well as longer intensive care unit (ICU) length of stay (LOS) [4]. When disorders of fluid homeostasis are present, such as diabetes insipidus (DI), achieving optimal fluid balance may be difficult. This is because of the high-volume urine output that can be seen with poorly controlled central and nephrogenic DI. Thiazide diuretics have been shown to be a safe and effective method of achieving fluid homeostasis in children with diabetes insipidus [5]. Their effectiveness is thought to be the result of increased sodium excretion leading to a reflexive uptake of sodium and water within the proximal renal tubule decreasing urine output [5]. There are few data on the use of other classes of diuretics to aid in the management of fluid and electrolyte disorders in patients with DI. Furthermore, no reports exist concerning the successful management of pediatric patients with DI and respiratory failure secondary to acute SARS-CoV-2 infection (COVID-19). We discuss here a unique case of a critically ill pediatric patient with hypoxemic respiratory failure from acute COVID-19 infection and previously diagnosed central DI who had severe fluid and electrolyte abnormalities. Homeostasis was achieved with the use of IV Furosemide in conjunction with a Vasopressin infusion.

## 2. Case Presentation

We present an 11-year-old female with complex medical history consisting of developmental delay, septo-optic dysplasia, central DI, panhypopituitarism including central adrenal insufficiency, hypothyroidism, and epilepsy. Her weight at presentation was 49.5 kg. She presented to the emergency department (ED) of a tertiary care academic medical center with impending respiratory failure (approximately two months after the first confirmed case of SARS-CoV-2 within the United States). She became ill eight days before presenting to the ED with fever. At that time, she was taken to a local urgent care facility where she was diagnosed with an acute otitis media. She was started on PO amoxicillin for an anticipated 10-day course of therapy. She was also given stress-dosed hydrocortisone (30 mg PO, twice daily) given her underlying panhypopituitarism. During the subsequent days, there was no reported change in oral intake or urine output (UOP). Her fevers initially improved; however, they never fully resolved. Three days prior to presentation, the patient experienced increased frequency and temperature of fevers. She again presented to a local urgent care facility where a nasopharyngeal swab was positive for the SARS-CoV-2 virus (via the PCR test). Within a few days, she developed a persistent, nonproductive cough, increased work of breathing, and shortness of breath, prompting the evaluation in the ED. Initial vital signs were remarkable for rectal temperature of 40.8°C, heart rate of 163 beats per minute, respiratory rate of 34 breaths per minute, and oxygen saturation of 89%. Physical exam revealed an alert female in a moderate amount of respiratory distress as evidenced by tachypnea and intermittent grunting. Dry oral mucosa was noted indicating a mild amount of dehydration. The remainder of the physical exam was unremarkable. Laboratory results were remarkable for hyperosmolar hypernatremia (serum sodium of 156 mmol/L and serum osmolality of 321 mOsm), mild hyperglycemia (serum glucose of 146 mg/dL), and elevated ESR (21 mm/hr), CRP (2.3 mg/dL), and procalcitonin (6.9 ng/dL). Her respiratory distress acutely worsened within hours of her ED arrival, and she was intubated for acute hypoxic and hypercarbic respiratory failure (immediate postintubation arterial blood gas with pH 7.20, PCO2 78, PO2 132, HCO3 30, and BD 1.7).

**Figure 1:**
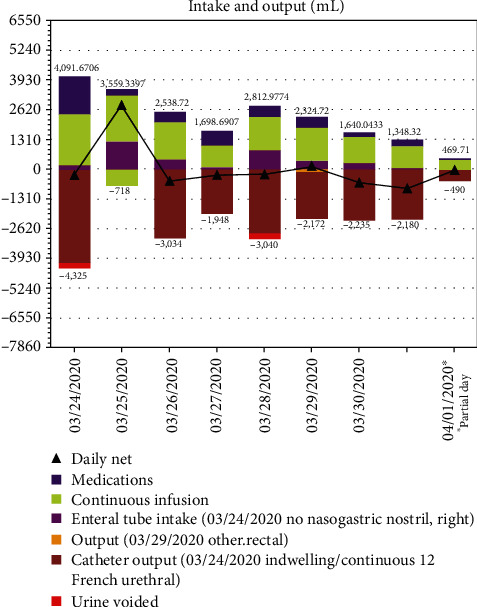
Overall fluid balance for the first week of PICU hospitalization as demonstrated by bar graphs of daily intake and output totals. The composition of fluids is depicted in color. Daily net fluid balance is depicted by triangles. Note the large net positive fluid balance on day 2 of hospitalization (3/25) with achievement of near net balance after initiation of Furosemide on day 3 (3/26).

**Figure 2:**
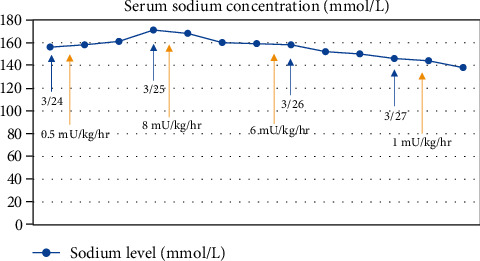
Serum sodium levels throughout first few days of admission. Blue arrows represent dates of hospital stay. Note the peak value of 171 mmol/L on day 2 of admission (3/25) with eventual decline after initiation of Furosemide with Vasopressin. Concurrent Vasopressin dosing is evidenced by yellow arrows. Note the initiation at 0.5 mU/kg/hr on 3/24, with a maximum infusion rate of 8 mU/kg/hr on 3/25, and eventual decline in dosing starting 3/26.

The patient was admitted to the pediatric intensive care unit (PICU). She was transitioned from her home regimen of subcutaneous desmopressin to a 0.5 mU/kg/hr Vasopressin infusion. Review of her baseline condition revealed that her DI was well controlled on her home regimen. She was continued on stress-dosed steroids (methylprednisolone 20 mg q6h IV). The patient had acute lung injury (SaO_2_/FiO_2_ = 120). Mechanical ventilator settings were adjusted to minimize ventilator-induced lung injury. Arterial and central venous lines were placed to promote more accurate determination of oxygenation (PaO_2_/FiO_2_ ratios) and venous monitoring. She was started on ceftriaxone, azithromycin, and hydroxychloroquine in addition to receiving one dose of tocilizumab. Ceftriaxone was used to treat presumed bacterial superinfection based on the severity of the patient's clinical status. At the time of the patient's presentation, our institutional protocol for the treatment of severe COVID-19 infection was based on current literature suggesting that the combination of azithromycin and hydroxychloroquine may achieve antiviral and anti-inflammatory effects against SARS-CoV-2 [6]. Additionally, at this time, tocilizumab was suggested to have beneficial effects against “cytokine storming” in SARS-CoV-2 patients [7]. The patient had hypotension that was unresponsive to fluid resuscitation. An epinephrine infusion was started to maintain adequate blood pressures. Given the patient's underlying conditions and critical state, chemistry panels were drawn every six hours on day one of admission. These panels quickly revealed a steady worsening of hypernatremia to a maximum value of 171 mmol/L associated with increasingly abundant UOP of more than 4 L on the first day of hospitalization (Figures 1 and 2). These changes occurred despite a maximum Vasopressin infusion rate of 8 mU/kg/hr along with appropriate, simultaneous adjustments in both the sodium concentration and rate of administered IV fluids.

By the second day of hospitalization, a net positive fluid balance of 2.8 L was noted and severe hypernatremia persisted (serum sodium of 160 mmol/L). Her moderate ARDS continued (PaO_2_/FiO_2_ = 132) [8]. When the hypernatremia persisted into the third day of hospitalization, a trial of Furosemide (10 mg IV) in addition to decreasing the Vasopressin dose from 8 mU/kg/hr to 6 mU/kg/hr was attempted. After five doses of IV Furosemide over a 48-hour period along with reduction in the Vasopressin dose, successful diuresis along with resolution of hypernatremia was achieved (net balanced fluid balance of -497 mL and reduction in serum sodium to 138 mmol/L, respectively) (Figures 1 and 2). Her ARDS gradually improved. The patient was extubated on hospital day 10. She remained hospitalized in the PICU for a total of 29 days during which time she was weaned from supplemental oxygen and was gradually weaned from sedative medications. She had normal serum sodium levels and UOP throughout the remainder of her PICU stay. She was discharged to her home with an outpatient subspecialist follow-up.

## 3. Discussion

The present case not only demonstrates the importance of adequate fluid balance in achieving optimal outcomes for a critically ill child with moderate ARDS but also highlights a novel use of Furosemide, a loop diuretic, in the management of DI. Fluid management in DI involves both management of fluid removal balanced with the maintenance of normal electrolyte concentrations. Overzealous treatment of central DI with ADH may result in water retention and iatrogenic hyponatremia, while insufficient treatment may result in dehydration and hypernatremia. In this case, the combination of IV Furosemide with a Vasopressin infusion promoted the excretion of sodium with a controlled UOP.

Mechanically ventilated patients are not the only population prone to the deleterious effects of fluid overload (FO). Studies have demonstrated that in various subsets of critically ill pediatric patients, including those not experiencing respiratory failure, fluid overload may lead to worse outcomes [2]. The exact degree of FO that yields these outcomes is unknown; however, 10% FO is usually when aggressive intervention is considered. This inconclusiveness has been thought to be a result of the lack of standardization of a method to calculate FO in both pediatric and adult patients [2]. Using the commonly used Goldstein et al.'s FB method [2], our patient had a maximum FO of 8.6% by day one of hospital stay.

Thiazide diuretics are a safe and effective method of treating pediatric patients with both central and nephrogenic DI [5]. While there are several proposed mechanisms, thiazide diuretics are thought to paradoxically slow UOP. Thiazides initially inhibit sodium uptake in the distal convoluted tubule yielding increased UOP. This diuresis leads to extracellular volume contraction, a decrease in the glomerular filtration rate, and, as a result, an uptake of sodium in the proximal convoluted tubule slowing further UOP [9]. Another proposed mechanism for their success involves the upregulation of aquaporin channels within collecting ducts. Aquaporin channels are downregulated in cases of both central and nephrogenic DI. Recent studies have shown that administering thiazide diuretics under these conditions results in an upregulation of aquaporin channels that further regulates UOP [10]. As a result of these effects, the UOP and therefore overall fluid balance become normalized.

It has been well demonstrated that many individuals infected with SARS-CoV-2 have experienced critical illness requiring prolonged ICU LOS and invasive mechanical ventilation [11]. Another complication that has been increasingly appreciated is acute kidney injury (AKI) [12, 13]. This seems to be attributed not only as secondary to hemodynamic and respiratory instability within these patients but also to direct viral insult on the proximal convoluted tubules and glomerulus [12–14]. While most children experience an asymptomatic or mild disease course with SARS-CoV-2 infection, critical illness still occurs and can be significant [15]. The risk of critical illness in pediatric patients may be increased especially in those with comorbidities [16]. Panhypopituitarism, as in this patient, would be expected to alter a patient's immune and homeostatic responses to an overwhelming viral infection making her possibly more susceptible to severe illness.

To our knowledge, this is the first report of a loop diuretic used in conjunction with a Vasopressin infusion achieving a desirable clinical effect in a child with DI and moderate ARDS [17]. Moreover, this effect was seen in a child with multiple comorbidities and acute COVID-19. The effect of Furosemide in our patient occurred robustly after only five doses. One putative mechanism for the additional action of Furosemide may be that the drug enhanced naturesis in a kidney in which appropriate water regulation had been restored by titration of exogenous Vasopressin. We recognize that a limitation of this report is that the mechanism(s) of the action of Furosemide in this case may remain imprecise.

## Data Availability

The data used to support the findings of this study are included within the article.
